# Deep sampling of the *Palomero *maize transcriptome by a high throughput strategy of pyrosequencing

**DOI:** 10.1186/1471-2164-10-299

**Published:** 2009-07-06

**Authors:** Julio C Vega-Arreguín, Enrique Ibarra-Laclette, Beatriz Jiménez-Moraila, Octavio Martínez, Jean Philippe Vielle-Calzada, Luis Herrera-Estrella, Alfredo Herrera-Estrella

**Affiliations:** 1 Laboratorio Nacional de Genómica para la Biodiversidad, Cinvestav Campus Guanajuato, Km 9.6 Libramiento Norte, carretera Irapuato-León. 36821, Irapuato, Gto, Mexico; 2 Boyce Thompson Institute for Plant Research, Ithaca NY, 14853, USA

## Abstract

**Background:**

In-depth sequencing analysis has not been able to determine the overall complexity of transcriptional activity of a plant organ or tissue sample. In some cases, deep parallel sequencing of Expressed Sequence Tags (ESTs), although not yet optimized for the sequencing of cDNAs, has represented an efficient procedure for validating gene prediction and estimating overall gene coverage. This approach could be very valuable for complex plant genomes. In addition, little emphasis has been given to efforts aiming at an estimation of the overall transcriptional universe found in a multicellular organism at a specific developmental stage.

**Results:**

To explore, in depth, the transcriptional diversity in an ancient maize landrace, we developed a protocol to optimize the sequencing of cDNAs and performed 4 consecutive GS20–454 pyrosequencing runs of a cDNA library obtained from 2 week-old *Palomero Toluqueño *maize plants. The protocol reported here allowed obtaining over 90% of informative sequences. These GS20–454 runs generated over 1.5 Million reads, representing the largest amount of sequences reported from a single plant cDNA library. A collection of 367,391 quality-filtered reads (30.09 Mb) from a single run was sufficient to identify transcripts corresponding to 34% of public maize ESTs databases; total sequences generated after 4 filtered runs increased this coverage to 50%. Comparisons of all 1.5 Million reads to the Maize Assembled Genomic Islands (MAGIs) provided evidence for the transcriptional activity of 11% of MAGIs. We estimate that 5.67% (86,069 sequences) do not align with public ESTs or annotated genes, potentially representing new maize transcripts. Following the assembly of 74.4% of the reads in 65,493 contigs, real-time PCR of selected genes confirmed a predicted correlation between the abundance of GS20–454 sequences and corresponding levels of gene expression.

**Conclusion:**

A protocol was developed that significantly increases the number, length and quality of cDNA reads using massive 454 parallel sequencing. We show that recurrent 454 pyrosequencing of a single cDNA sample is necessary to attain a thorough representation of the transcriptional universe present in maize, that can also be used to estimate transcript abundance of specific genes. This data suggests that the molecular and functional diversity contained in the vast native landraces remains to be explored, and that large-scale transcriptional sequencing of a presumed ancestor of the modern maize varieties represents a valuable approach to characterize the functional diversity of maize for future agricultural and evolutionary studies.

## Background

Sequencing and analysis of expressed sequence tags (ESTs) has been a primary tool for the discovery of novel genes and for annotation of genomic sequences in plants. ESTs provide large-scale characterization of mRNA populations through single-pass sequencing of cDNA. In crop species with a highly repetitive genome like maize, EST sequencing represents a rapid and cost-effective method for analyzing the transcribed region of the genome, allowing a distinction between functional genes and pseudogenes. ESTs can be used for other functional genomic projects including gene expression profiling, microarrays, molecular markers and physical mapping. Sequencing of ESTs from a non-normalized cDNA library using a high throughput approach could be useful for the quantitative assessment of transcript abundance and also for the discovery of novel transcribed sequences. In addition, ultra-deep sequencing of a non-normalized cDNA library could overcome the high sequence redundancy rates that the library might present.

Quantitative estimates of gene expression are also possible with large number of ESTs derived from diverse libraries [1]. Other high throughput approaches for quantitative and qualitative genome-wide gene expression profiling are Serial Analysis of Gene Expression (SAGE) [2] and Massively Parallel Signature Sequencing (MPSS) [3]. SAGE has been largely used in animal systems and more recently SAGE collections for several plant species have been made available [4-7]. In contrast, MPSS has been more widely used in plants than in animal species [8,9].

Large-scale pyrosequencing of cDNAs offers a unique and an alternative opportunity to deeply explore the nature and complexity of a given transcriptional universe. Currently, one GS20–454 sequencing run produces a minimum of 200,000 reads with an average length of 100 nt. Applications of the 454 technology in plants include the sequencing of barley's BACs [10], *Arabidopsis thaliana *miRNAs [11] and cDNA libraries of *Medicago truncatula *[12], *A. thaliana *[13] and the shoot apical meristem of maize [14]. Although these efforts have produced a large amount of valuable transcriptional information, the procedure has not yet been optimized for the sequencing of cDNAs, and the amount of sequencing runs or GS20–454 reads that are necessary to reach full coverage or "near identity saturation" of a target transcriptome remains to be determined. An estimation of these types of representational parameters is important for large-scale EST projects that rely on 454 technology for large-scale transcriptional analysis.

Mexico is considered the center of origin and domestication of maize. With no less than 59 native landraces and many distinct environmental adaptations, Mexican germplasm has been essential to harness important traits for crop improvement. *Palomero Toluqueño is *a landrace of the Central and Northern Highlands Group characterized by short plants with frequent tassel branches, small conically shaped ears, a weakly developed root system, and pubescent leaf sheaths often pigmented by anthocyanins. It is one of several ancient landraces that are believed to have spread from the Pacific Coast to Northern areas of Mexico, contributing to the emergence of popcorn elite cultivars in the USA [15].

As part of a genomic platform for the systematic exploration of landrace genetic diversity, we analyzed over 1.5 Million quality-filtered reads generated by 4 consecutive pyrosequencing runs of a single cDNA library derived from 2 week old plants of *EDMX2233 Palomero Toluqueño *maize, and compared them to publically available ESTs, and Maize Assembled Gene Islands (MAGIs) from the B73 maize inbred line. MAGIs are genomic sequence assemblies from regions that are enriched in transcriptionally active units [16]. This collection of 454 quality-filtered reads was sufficient to find transcripts corresponding to 50% of public maize ESTs. Comparisons to the MAGIs revealed that 11% of them align with our collection of *Palomero *sequences. We estimate that 5.67% (86,069 sequences) do not align with public ESTs or annotated genes and potentially represent new maize transcripts. Our results indicate that recurrent pyrosequencing is necessary to attain a thorough representation of the transcriptional universe present in a single cDNA sample, suggesting that large-scale transcriptional sequencing of native germplasm will emerge as an important tool to characterize the functional diversity of maize, as well as the identification of relevant genes for particularly interesting agronomic traits.

## Results

### Generation and Sequencing of the *Palomero *cDNA Library

A cDNA library was generated from total RNA extracted from young aerial and root tissues of a Mexican maize landrace as described in Material and Methods. We used a procedure for preparation of the maize cDNA library that overcomes possible bias that may occur when sequencing short sequences of DNA by 454 technologies. For library construction, 3'-enrichment of sequences was avoided by using random primers rather than a poly(T) primer during a second round of cDNA synthesis; the resulting cDNA sample was sheared by nebulization and end-repaired before ligating the 454 sequencing adapter. It is expected that synthesis of cDNA using oligo-dT primers will yield sequences that are 3'-enriched relative to the entire transcriptome, resulting in sequences frequently containing polyadenylated tails that significantly reduce the length of informative reads.

Four runs of the cDNA sample produced 1,526,880 reads with an average length of 100.51 nucleotides and a total length of 153.47 Mb. All reads were filtered to remove poly A/T, low quality sequences and those shorter than 50 nt using the SeqClean program. After trimming, the 1,526,880 raw sequences were reduced to 1,517,878 (99.41%) high quality sequences with an average length size of 100.38 nt and a total length of 152.37 Mb (Table 1). Only 1.2% of raw sequences were trimmed for eliminating polyadenylated tails, and only 0.59% of raw sequences were removed due to their length (shorter than 50 nt) or because of their low quality score. This is in contrast to a previous report on the utilization of GS20–454 sequencing for large-scale transcriptional analysis of a cDNA library from *Arabidopsis*. Two sequencing runs yielded 555,326 raw reads with a mean length of 108 nt that was reduced to 89.2 nt after quality control using SeqClean, whereas the removed sequences represented 2.4% of the total raw reads [13]. The reduced amount of low quality sequences in our library, and the average length size reduction of 0.13 nt in the sequences after trimming is a significant improvement of the entire sequencing process using the 454 technology. The low amount (1.2%) of the ESTs containing a poly A/T tail was expected as the filter for these homopolymers was applied during the cDNA library construction by the use of random primers. In addition, alignment of reported maize ESTs that matched with several 454 reads shows no bias of the 454 reads towards any end of a corresponding EST (i.e. 3' or 5' region). Furthermore, we found that only 0.04% of the *Palomero *reads have a match to maize tRNAs and plant small nucleolar RNAs (snoRNAs). The use of random primers combined with sample nebulization significantly improved the percentage of informative sequences as well as their length and quality, showing that these modifications are crucial to obtain high quality sequences representing a wide transcriptional universe.

**Table 1:** Statistics of the high quality reads from four GS20–454 sequencing runs of the *Palomero *cDNA library and coverage of the maize unigenes from the NCBI (UniGene) and TIGR (ZMGI) databases after each sequencing run.

**Run GS20–454**	**Num. reads (Mb)**	**Avg length of read (nt)**	**Cumulative runs**	**ZMGI (N = 115744 seq)**		**NCBI (N = 55327 seq)**	
				
				**Matches (%)**	**Increase (%)**	**Matches (%)**	**Increase (%)**
1	367391 (37.09)	100.94	1	36.42	0	37.89	0
2	367699 (37.04)	100.73	+ 2	43.58	7.16	44.12	6.23
3	394851 (39.73)	100.62	+ 3	47.83	4.25	47.74	3.62
4	387937 (38.99)	100.50	+ 4	50.42	2.59	50.00	2.26
Total	1517878 (152.37)	100.38					

### Analysis of High Quality Reads from GS20–454 Runs and Comparison to Gene Index and UniGene Databases

The number of individual reads between each 454-sequencing run showed a notorious homogeneity (Additional file 1). After trimming we had a minimum of 367,391 (37.09 Mb) and a maximum of 394,851 (39.73 Mb) reads per run in all four sequencing runs (Table 1). This represents a considerable increase in the average number of reads reported so far for a 454 run in cDNA libraries from plants. For instance, one single 454 run of a *Medicago *[12] and maize [14] cDNA library resulted in 252,000 (23 Mb) and 260,000 high quality reads, respectively. In addition, two sequencing runs of an Arabidopsis cDNA library yielded 541,852 ESTs [13]. Here, we obtained 40% more high quality sequences per run than those reported previously for plants, indicating that our sequencing-by-synthesis (SBS) approach represents an efficient strategy to generate large amounts of ESTs.

**Figure 1:**
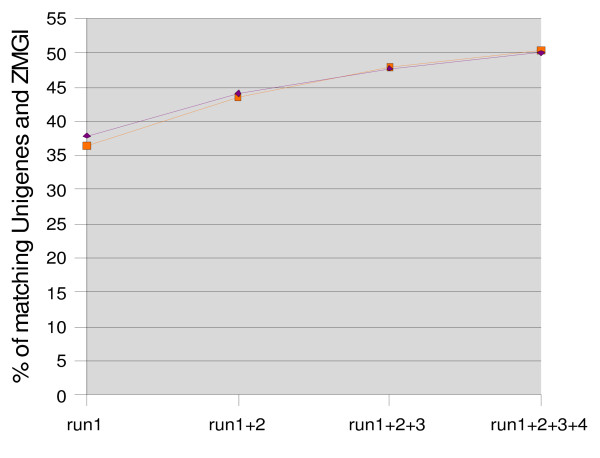
**Percentage of matching Unigenes from NCBI (purple) and TIGR (red) after four GS20–454 sequencing runs**. A comparison by BLAST (e-value < 9e-07) was performed with the *Palomero *454 sequences against the NCBI (UniGene) and TIGR (ZMGI) databases.

A BLAST-based search against the NCBI maize UniGene (UniGene Build number 61, January 18^th ^2007) set revealed that 27,664 expressed genes (roughly 50% of the UniGene database) are represented in our collection of unassembled *Palomero *transcript reads. Although these UniGene set can be considered the minimum number of expressed genes represented in our transcript collection, it is likely an under-estimation of the universe of transcripts present in a 2 week old plant. The *Zea mays *Gene Index (ZMGI) database from TIGR contains 115,744 assemblies and singletons derived from a wide variety of maize ESTs libraries (date of release; November 17^th^, 2006). Despite a possible redundancy of gene representation within the ZMGI assembly [17], this database can be used to estimate the fraction of the maize transcriptome covered by our GS20–454 collection of *Palomero *transcripts. A BLAST comparison of all sequences generated in a single run showed that 36.42% of ZMGI sequences align with at least one GS20–454 sequence (Table 1). This proportion increased to 50.42% when the comparison included all sequences generated after 4 runs. Whereas the proportion increased in 7.16% following a second GS20–454 run, the fourth run increased the ZMGI gene representation by only 2.59%, indicating that a plateau of gene representation was reached following the third GS20–454 run (Figure 1). A similar comparison conducted against the UniGene dataset yielded similar results (Table 1). Overall, these results suggest that approximately 55% of genes or ESTs contained in these databases are represented in the 2 week old plant GS20–454 *Palomero *transcript collection.

### Comparison to Maize Assembled Genomic Islands (MAGIs)

To discover previously identified genomic sequences that could have new evidence of expression, we compared the Maize Assembled Genomic Islands (version 4, ) [16] to our *Palomero *transcript collection using BLAST. A total of 77,045 MAGIs aligned to at least one GS20–454 transcript sequence of *Palomero *(compared to 74,403 MAGIs that aligned to at least one NCBI maize EST; Table 2), providing evidence that these MAGIs contain at least portions of expressed genes. Overall, these 77,045 aligned to 89.5% of all GS20–454 sequences. The remaining 10.5% of GS20–454 sequences that did not have a representation in at least one MAGI could represent genes that have not yet been sequenced in B73, but also genes specific to the *Palomero *landrace, or *Palomero *genes having poor homology to a possible B73 ortholog. From all 77,045 MAGIs with evidence of expression after four GS20–454 runs, 34,752 (45.1%) did not have prior expression evidence from the alignments to the 903,624 NCBI maize ESTs. Table 3 shows the number of MAGIs that did not have prior expression evidence in the NCBI maize ESTs for each GS20–454 run. 15,898 MAGIs showed novel expression evidence with sequences from a single run, less than 50% of the 34,752 MAGIs that showed novel expression evidence with all 4 sequencing runs. Therefore, increasing the number of 454 sequencing runs shows a significant increase on the number of novel genomic sequences matched with expressed sequence tags, indicating expression evidence for such genome regions.

**Table 2:** Comparison of the number of the NCBI maize ESTs and *Palomero *GS20–454 ESTs aligning by BLAST with the MAGIs.

	**NCBI ESTs**	**454 ESTs**
Num. seq.	903624	1517878
% Hits	98.32	89.52
% No hits	1.68	10.48
Num. MAGIs matched (N = 727781 seq)	74403	77045
Distinct MAGIs matched	32110	34752

**Table 3:** Number of unique MAGIs with a match to the *Palomero *GS20–454 ESTs that did not have prior expression evidence in the NCBI maize ESTs.

**Runs 454**	**MAGIs matching the GS20–454 ESTs**	**MAGIs matching both NCBI ESTs and GS20–454 ESTs**	**Unique MAGIs matching the GS20–454 ESTs**
1	47278	31380	15898
+ 2	60807	37034	23773
+ 3	70348	40296	30052
+ 4	77045	42293	34752

### Gene Discovery and Characterization of Novel Transcripts

To determine the number of potential novel genes found in the *Palomero *transcript collection, the total GS20–454 high quality reads were compared to the unassembled NCBI maize ESTs (from January 18th 2007), the MAGIs version 4, the maize chloroplast and mitochondria genomes and the MAGI repeats (version 3.1). Using an e-value < 9e-07, 87.5% of the GS20–454 sequences aligned with the existing NCBI maize ESTs, and 89.52% aligned with the genomic sequences of MAGIs (Table 4 and Additional file 2). Interestingly, only 1.75% and 2.7% of all GS20–454 sequences aligned to maize organelles and MAGI repeats, respectively (Table 4). In the case of the comparison to the EST collection of NCBI, 87.5% of all GS20–454 sequences matched directly by BLAST to a specific maize transcript, whereas 189,594 (12.5%) did not match with any reported maize transcribed sequence. Thus, using a relatively high level of stringency, 12.5% of the GS20–454 sequences potentially identify novel maize transcripts. This percentage was reduced to 5.67% (86,069 GS20–454 sequences) after a BLAST alignment to MAGIs, MAGI repeats and maize organelle databases, yielding the most conservative estimation of the proportion of novel transcripts that are represented in our *Palomero *collection.

**Table 4:** Percentage of the *Palomero *GS20–454 sequences from four sequencing runs that matched by BLAST to maize databases and those that did not align (e-value < 9e-07) to any maize database.

**Database**	**% of 454 reads matching**	**% of 454 reads** **without a match**
NCBI_ESTs (903624 seq)	87.50	
MAGI.4 (727781 seq)	89.52	5.67 (86,509)
Zm_organelle (1 ch + 4 mit)	1.75	
MAGI_repeats (13564 seq)	2.70	

**Figure 2:**
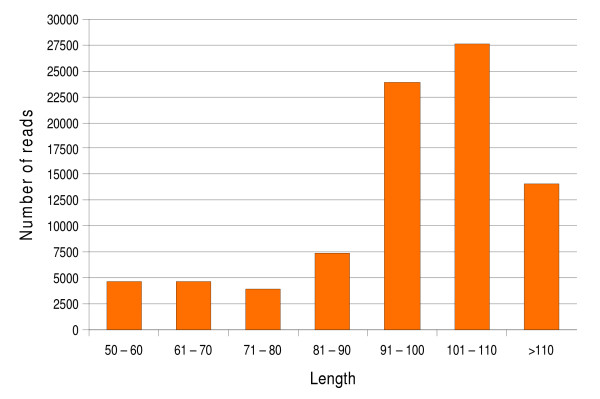
**Histogram of the length (in nucleotides) distribution of the 86,069 *Palomero *GS20–454 sequences from the four sequencing runs that did not align by BLAST (e-value < 9e-07) with any maize database**.

Figure 2 shows a histogram of the size distribution of these 86,069 sequences. In addition, we calculated the % of GC of this set of sequences and compared it to that of a set of 88,299 random sequences from the same *Palomero *EST collection. The potential novel transcripts had 50% of GC and an average length of 96.96 nt, whereas the set of random sequences had 51% of GC and an average length of 97.02 nt, indicating that there are no significant differences in the GC fraction and length of both sets of sequences. The potential novel sequences were then searched by BLAST against the TIGR Plant Transcript Assemblies database (Plantta), which contains all the expressed sequences from all plant species for which more than 1,000 ESTs or cDNA sequences are publicly available [18]. 13,464 sequences had a match to this database, averaging 2.3 sequences by gene locus (5,854 genes), of which 32.6% are annotated as hypothetical proteins. These results suggest that there are a significant number of maize genes that are not present in the public maize EST databases that are transcribed and have an orthologous gene in other plant species. After this analysis still 72,305 sequences did not align to any known plant transcript. A comparison by BLASTX to the non-redundant protein database (NR) from NCBI of these sequences revealed similarity of additional 7,997 sequences to a given protein (averaging 1.7 sequences by gene locus), most of them (> 60%) cataloged as hypothetical proteins. Thus, 64,308 ESTs failed to align to any known protein, probably representing transcripts from non-coding genomic sequences.

### Representation of Emblematic Maize Genes

To assess the representation of different well-known maize genes in our *Palomero *transcript collection, we aligned all GS20–454 sequences with those corresponding to 18 maize genes shown in table 5. This same table indicates the number of GS20–454 reads that align with genes in stretches of 30, 29, 28 and 27 nucleotides without gaps (100%, 96.6%, 93.3% and 90% of identity, respectively). Genes that are highly represented but seem to be more divergent in sequence are for instance ramosa2 and c1. Poorly or not represented genes are floricaula/leafy-like2 (fl2), knotted1-like homeodomain protein liguleless3 (lg3), ramosa1 (ra1) and teosinte branched1 (tb1). Low expression of fl2, a gene involved in flower development [19] was expected due to the nature of the tissue used for the cDNA synthesis (two week-old plants). However, low representation of other genes such as *tb1 *was unexpected due to its known role in regulating the formation of secondary axillary branches in maize [20]. These results suggest that a complex regulation of the active transcriptome makes it difficult to predict the presence of certain transcripts based solely in the nature of the tissue used.

**Table 5:** Representation of emblematic maize genes in the *Palomero *GS20–454 cDNA library.

				**Number of reads**^a^			
				
	**Gene Name**	**Length (nt)**	**Status**	**90%**	**93.3%**	**96.6%**	**100%**
floricaula/leafy-like2	fl2	2921	partial CDS	1	---	---	---
barren stalk1	ba1	4570	complete CDS	31	15	3	---
rough sheath2	rs2	1420	complete CDS	7	6	2	1
alcohol dehydrogenase1	adh1	1389	partial CDS	59	59	56	34
alcohol dehydrogenase2	adh2-n	3535	complete CDS	55	48	46	28
knotted1-like homeodomain protein liguleless4b	lg4b	1123	partial CDS	6	4	4	3
knotted1-like homeodomain protein liguleless3	lg3	1537	complete CDS	---	---	---	---
indeterminate gametophyte1	Ig1	5431	complete CDS	31	13	9	5
ramosa2	ra2	6032	complete CDS	111	45	22	4
ramosa1	ra1	1519	complete CDS	---	---	---	---
teosinte glume architecture1	tga1	3219	complete CDS	13	9	---	---
P gene; transposon		7753	complete CDS	349	94	23	2
knotted-1	kn-1	1627	complete CDS	91	37	14	10
teosinte branched1	tb1	3188	partial CDS	---	---	---	---
indeterminate growth1	id1	1625	complete CDS	4	2	2	2
c1 locus myb homologue	c1	4059	complete CDS	85	41	5	---
Bronze2	bz2	2948	complete CDS	19	15	7	---
fertilization independent endosperm2	fie2	1171	complete CDS	17	15	15	10

^a^A criterion of 90 to 100% identity was used to consider a hit valid

### Assembly of GS20–454 Transcripts and Quantitative Assessment of Transcriptional Abundance

Assembly of the total GS20–454 raw sequences was performed using the 454 commercial software utilities. A total of 1,135,969 (74.4%) reads were assembled into 65,493 contigs, 134,888 (8.8%) reads were classified as singletons and 240,535 (15.8%) sequences were classified as "repeats" on the basis of their over-representation that is likely to reflect abundant transcripts. Sequences in this latter category include highly expressed transcripts that are generally difficult to assemble. We found that 89% of these sequences have a hit to the ZMGI, averaging 7.9 reads per gene locus, whereas a similar analysis with the contigs and singletons averaged 1.7 sequences per gene locus and 84% and 48% of the sequences have a hit to ZMGI, respectively. In addition, 89.5% of the total GS20–454 reads aligned to ZMGI. These data indicate that the unassembled sequences represent valuable information that cannot be excluded from the global analysis of the *Palomero *ESTs, and justify the use of individual GS20–454 reads for the coverage analysis of public databases as described in this work.

Analyses performed with the 65,493 assembled contigs included transcript abundance estimation and a survey of the contribution of our assemblies to the length of the sequences in the ZMGI. For the latter, we compared the 65,493 assembled contigs to the ZMGI database and estimated the number of GS20–454 contigs having a sequence length larger than the aligned ZMGI sequence. From 54,743 contigs with a significant match to ZMGI, we only found 468 that were larger than the aligned ZMGI sequence. In addition, a TGICL-dependent assembly of all 86,069 GS20–454 sequences candidate to represent novel transcripts resulted in 9,040 contigs and 55,146 singletons, suggesting that most of these unique sequences represent rare transcripts.

Relative expression levels of known genes or ESTs can be approximately quantified by hybridization to microarrays; however, it is limited to genes that have been printed in the microarray, usually genes which sequence was previously determined or predicted based on genome annotation. To determine whether results of our high-throughput pyrosequencing approach reflect transcript abundance, we estimated relative abundance of several transcripts based on number of GS20–454 sequences assembled into a given contig and the length of that contig, according the following index:

Ra = N/L; where Ra, relative abundance; N, number of GS20–454 sequences per contig; L, length of the assembled contig.

The comparison of the 454 assembled contigs against the ZMGI was used to assign an annotated gene locus to the 454 assemblies. To test this transcript abundance estimation and to calculate a relative expression ratio, we performed quantitative real-time PCR (qPCR) of several 454 contigs. Sequences to be amplified by real-time PCR were chosen according to their relative differential abundance as estimated by the index described above. Primers were chosen to amplify a region of approximately 170 bp. Additional file 3 shows the set of primers designed for real-time PCR of 8 different contigs. We found a general correlation between transcript abundance estimation based on the 454 sequencing and the qPCR data. Table 6 shows a comparison of the relative abundance of several transcripts calculated by the formula described above and the cycle threshold (Ct) obtained by qPCR analysis. Lower Ct numbers are expected for highly abundant transcripts. For instance, the contig for TC327885 (Ra = 2.11) appeared six cycles earlier than the contig for TC327155 (Ra = 0.08). Although other approaches may be needed for accurately profile transcript abundance in a cDNA library, these results suggest that high-throughput sequencing-by-synthesis is useful to generate quantitative information of the transcripts.

**Table 6:** Comparison of transcript abundance of representative maize genes estimated by GS20–454 sequencing and qPCR.

**Gene locus matched**	**Accession (ZMGI)**	***Ra*454 reads**	***Ct *qPCR (mean ± SD)**
Similar to UP|LIRP1_ORYSA (Q03200) Light-regulated protein precursor	TC327885	2.11	11.7808 ± 0.09
Similar to UP|PSAE_HORVU (P13194) Photosystem I reaction center subunit IV, chloroplast precursor (PSI-E)	TC361477	0.74	13.1666 ± 0.05
UP|Q41754_MAIZE (Q41754) Ubiquitin	TC369342	0.49	14.6629 ± 0.10
UP|TBA6_MAIZE (P33627) Tubulin alpha-6 chain (Alpha-6 tubulin)	TC326717	0.32	14.0284 ± 0.03
Homologue to GB|BAD33626.1|50726105|AP005579 Polyubiquitin 2	TC342043	0.32	16.3357 ± 0.04
Homologue to UP|Q41772_MAIZE (Q41772) Cytosolic ascorbate peroxidase, complete	TC364641	0.22	16.5425 ± 0.08
Similar to UP|SODC2_MESCR (O49044) Superoxide dismutase [Cu-Zn]2	TC327155	0.08	17.9920 ± 0.08
Similar to UP|Q6YIH2_ORYSA (Q6YIH2) OsCDPK protein	TC327230	0.06	18.5091 ± 0.13

## Discussion

The development of pyrosequencing technologies (in particular 454 sequencing) has contributed to total sequence information available for several multicellular organisms. In the case of maize, a single GS20–454 run with cDNA amplified from shoot apical meristems of inbred line B73 resulted in ~261,000 ESTs that were sufficient to annotate more than 25,000 genomic sequences [14]. A similar approach was used to demonstrate that 454-based transcriptome sequencing of inbred lines allows high-throughput acquisition of gene-associated single nucleotide polymorphisms (SNPs) [21]. More recently, large-scale sequencing of 3'-UTR regions was used to resolve the expression of gene families, allowing a frequent distinction between alleles and gene family members [22]. Although these studies have demonstrated the value of large-scale pyrosequencing technologies when applied to the analysis of specific maize transcriptomes, an in-depth estimation of the overall transcriptional universe found at a specific developmental stage had not been previously carried out.

We performed 4 consecutive GS20–454 pyrosequencing runs of a single cDNA library obtained from seedlings of *Palomero Toluqueño *collected 2 weeks after germination, and generated the largest collection of maize transcripts corresponding to a single developmental stage. On average we obtained over 37 Mb per run and a total of 152.37 Mb of high quality sequence, and our overall coverage was sufficient to detect transcripts similar to at least 50% of all publically available ESTs present in the UniGene and ZMGI databases. The total number of ZMGI sequences that are represented in our transcript collection increased 14% between the first and the fourth pyrosequencing run; however, the fourth and last run only yielded an increase of 2.59%, indicating that despite the importance of increasing the number of sequencing runs in terms of statistical accuracy, the last run had little contribution to the overall coverage and the discovery of novel transcripts. This percentage is slightly increased when pyrosequencing reads are compared to the MAGI collection, suggesting that MAGIs might have an under representation of rare or low abundant transcripts. This is supported by the fact that increasing the number of 454 sequencing runs shows a significant increase on the number of novel genomic sequences matched with expressed sequence tags, providing expression evidence for such genome regions, which most probably represent genes or transcriptionally active non-coding regions with low levels of expression. Overall, our analysis suggests that 3 consecutive pyrosequencing runs are sufficient to obtain a representation of most of the transcriptome present in *Palomero *plantlets.

The phenotypic and molecular diversity of maize has been essential to harness important traits for crop improvement. On the basis of landrace germplasm, the activity of modern plant breeders gave rise to inbred lines currently used in hybrid production, causing significant improvements in yield, grain quality, resistance to biotic or abiotic stress, and maturity. A genome wide survey of gene content in B73 and Mo17 revealed that more than 20% of gene fragments examined in allelic contigs were not shared between these 2 inbred lines [23]; reasonable predictions anticipate that the genomic divergence between 2 landraces is far more important. Our results identified more than 86,000 sequences that represent novel transcripts that are expressed in *Palomero *plantlets, indicating that a large portion of the intrinsic transcriptional diversity present in native landraces remains to be explored. The discovery of this collection of novel transcripts suggests that many more should be present in different tissues and developmental stages, opening the possibility for large-scale efforts to characterize the transcriptional universe of genetically distinct native landraces.

When estimating transcriptional abundance of representative genes, we noticed a direct correlation between the number of reads corresponding to a transcript and its level of expression assessed by qPCR, indicating the possibility that in some transcriptional ranges, deep sequencing of cDNA samples could provide an accurate estimation of transcriptional abundance. It is likely that an increase in the number of pyrosequencing runs could enhance the accuracy of this type of quantitative estimations, as the number of pyrosequencing runs necessary for deep coverage of a given transcriptome will depend on the nature and the complexity of the sample. Overall, our results suggest that a systematic and detailed characterization of gene expression in maize using high-throughput technologies will generate useful information for the understanding of maize biology.

Access to large-scale landrace transcriptional sequences promise to become an invaluable source of polymorphic information for exploring maize natural variation and exploiting allele diversity and recombination. We expect that a renewed interest in landrace germplasm will emerge with the development of new initiatives to explore the functional diversity of maize.

## Conclusion

In conclusion, using an optimized protocol for pyrosequencing of a *Palomero *cDNA library we generated and analyzed the largest collection of maize transcripts corresponding to a single developmental stage. The *Palomero *sequences covered over 50% of all reported maize unigenes, and an estimated of 5.67% of the reads potentially represent new maize transcripts. Our results indicate that recurrent pyrosequencing is necessary to attain a thorough representation of the transcriptional universe present in a single cDNA sample, as well as for transcript abundance estimation in a non-normalized cDNA library. Finally, large-scale transcriptional sequencing of native landraces represents a valuable approach to characterize the functional diversity of maize.

## Methods

### Plant material

Seeds from *Zea mays Palomero *(accession# EDMX2233, CIMMYT, Mexico) were grown under greenhouse conditions for 2 weeks and then transferred to a dark room for two days before total RNA extraction.

### cDNA library construction

Total RNA was extracted with TRIZOL (Invitrogen) from whole 2 week old maize seedlings. cDNA synthesis was performed with 3.5 μg of total RNA using Message Amp-II kit (Ambion) following the protocol as recommended by manufacturers. Briefly, first strand cDNA synthesis was primed with T7 Oligo(dT) primers. After a second strand cDNA synthesis reaction, 5–10 ng of synthesized double-stranded cDNA were amplified by in vitro transcription and the resulted 5–7 μg of antisense RNA (aRNA) was purified using Qiagen RNAeasy columns (Qiagen). A second round of cDNA synthesis was performed using the aRNA as template. First and second strand cDNA synthesis were as described above except that random nonamers (Amersham) were used at the first strand synthesis. This procedure yielded about 4 μg of cDNA that were purified using the DNA Clear Kit for cDNA purification (Ambion). cDNA was nebulyzed to obtain fragments of 200–700 bp before sequencing.

### GS20–454 sequencing

Approximately 3 μg of sheared cDNA were used for GS20–454 sequencing. The cDNA sample was end-repaired and adapter ligated according to [24]. Streptavidin bead enrichment, DNA denaturation and emulsion PCR were also according to procedures previously described [24]. Four sequencing runs were performed in this library and resulted in 1,526,880 reads.

### Sequence analysis

Trimming of polyA/T and removing of low quality sequences from the raw 1,526,880 reads was performed using TIGR SeqClean software pipeline . Sequences shorter than 50 bp after processing were excluded from the analysis. This resulted in 1,517,878 high quality reads. For assembly, the 454 Newbler software and the TIGR Gene Indices clustering tools (TGICL) [25] were used.

Stand-alone BLAST software [26] was obtained from the National Center for Biotechnology Information (NCBI, ). The high quality GS20–454 sequences were compared by BLAST with 903,624 unassembled maize ESTs from GenBank (downloaded in January 2007), 55,327 maize UniGenes from GenBank (Build number 61, January 18^th ^2007), 727,781 contigs and singletons from MAGI version 4 and the MAGI Cereal Repeat database v 3.1 , 115,744 maize sequences from the TIGR Gene Indices downloaded in November 2006 (ZMGI release 17, ), and the maize chloroplast (GenBank accession no. X86563) and mitochondrial (GenBank accession no. DQ645537*Zea luxurians*; AY506529*Zea mays *strain NB; DQ645539*Zea mays *subsp. parviglumis; DQ645538*Zea perennis*) genomes. A database containing 1561 maize tRNAs [27] and 421 plant snoRNAs  was used to search for these RNAs in the GS20–454 sequences. Other databases used in this study are the TIGR Plant Transcript Assemblies database (Plantta) [18] and the non-redundant protein database (NR) from NCBI . Several local MySQL databases were built to store all relevant information of BLAST analyses. Perl scripts were used to retrieve sequences from the *Palomero *EST collection. Sequences of the assemblies of the 454-GS20 reads were deposited in the GenBank Transcriptome Shotgun Assembly (TSA) database under accession numbers EZ048883 – EZ114339.

### qPCR

Primers for qRT-PCR were designed to produce amplicons of about 170 bp (see Additional file 3). The reaction mixture for quantitative PCR was as follows: 10 μl of Sybr green master mix (Applied Biosystems), 3 μl of cDNA template (3 ng/μl) and 1 μl of each (10 μM) of the primers. The PCR program was as follows: One cycle at 95°C for 5 min, 40 cycles at 95°C each for 30 sec, at 65°C for 30 sec, 72°C for 40 sec. Melting curves for each product, starting from 60°C to 95°C at 0.2°C/sec, produced a single melting point. All the Ct values are averages of at least three repetitions.

## Authors' contributions

JCVA designed the experiments, performed genomic and bioinformatic analyses, and wrote the manuscript; EIL performed cDNA amplifications and qPCRs; BJM was responsible for GS20–454 sequencing; OMV contributed to the design of the bioinformatic analyses; JPVC contributed to the design of the experiments and assisted in the writing of the manuscript; LHE contributed to the design of the experiments and managed funding; AHE initiated the project, contributed to the experimental design and assisted in the writing of the manuscript. All authors read and approved the final manuscript.

## Supplementary Material

Additional file 1 **A histogram showing the linear increase of the number of sequences after four GS20–454 runs**. The number of high quality sequences is plotted with the number of runs. The total of the sequences generated by all the four sequencing runs are depicted. Click here for file

Additional file 2 **Number of matching NCBI maize ESTs and MAGIs to the *Palomero *GS20–454 sequences after each sequencing run**. This table summarizes the BLAST results of all the *Palomero *GS20–454 reads against the NCBI ESTs and MAGIs. Click here for file

Additional file 3 **Primers used in this study**. This table contains the sequence of primers used for qRT-PCR. Click here for file
