# Supplementary figures and images for: Deep sampling of the Palomero maize transcriptome by a high throughput strategy of pyrosequencing
Source: BMC Genomics. 2009 Jul 6;10:299. doi: 10.1186/1471-2164-10-299 (PMC2714558; doi:10.1186/1471-2164-10-299)

## Slide 1
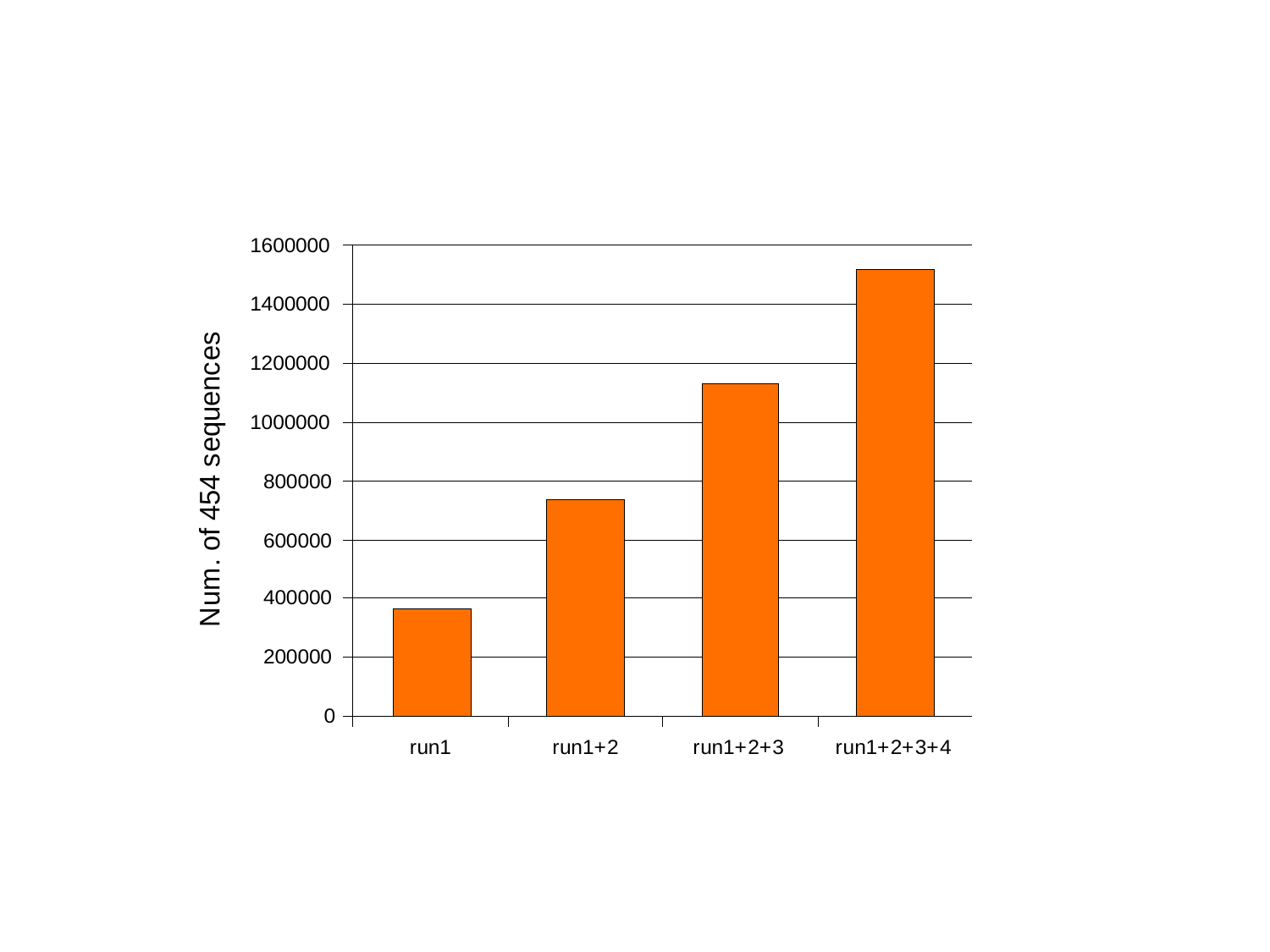

Supplement: Additional file 1 — A histogram showing the linear increase of the number of sequences after four GS20–454 runs. The number of high quality sequences is plotted with the number of runs. The total of the sequences generated by all the four sequencing runs are depicted. [file 1471-2164-10-299-S1.ppt]
