# Supplementary material for: Deep sampling of the Palomero maize transcriptome by a high throughput strategy of pyrosequencing
Source: BMC Genomics. 2009 Jul 6;10:299. doi: 10.1186/1471-2164-10-299 (PMC2714558; doi:10.1186/1471-2164-10-299)
Supplement: Additional file 2 — Number of matching NCBI maize ESTs and MAGIs to the Palomero GS20–454 sequences after each sequencing run. This table summarizes the BLAST results of all the Palomero GS20–454 reads against the NCBI ESTs and MAGIs. [file 1471-2164-10-299-S2.doc]

**Additional file 2. Number of matching NCBI maize ESTs and MAGIs to the Palomero GS20-454 sequences after each sequencing run.**

| **GS20-454 runs** | |  | **Matching**  **NCBI ESTs**  **(N=903624 seq)** | **Aligned**  **454 ESTs**  **(N=1517878 seq)** |  | **Matching**  **MAGIs**  **(N= 727781 seq)** | **Aligned**  **454 ESTs**  **(N=1517878 seq)** |
| --- | --- | --- | --- | --- | --- | --- | --- |
| **1** |  | | **101093**  **(11.19)^a^**  **138248**  **(15.3)**  **164106**  **(18.16)**  **181818**  **(20.12)** | **321701**  **(87.50)**  **643107**  **(87.50)**  **988766**  **(87.49)**  **1328284**  **(87.56)** |  | **47278**  **(6.5)**  **60802**  **(8.35)**  **70348**  **(9.66)**  **77045**  **(10.59)** | **329072**  **(89.57)**  **658266**  **(89.55)**  **1011842**  **(89.55)**  **1358742**  **(89.52)** |
| **1+2** |  | |  |
| **1+2+3** |  | |  |
| **1+2+3+4** |  | |  |

^a^: Parenthesis indicates percentage of the total number (N) of sequences
