# Supplementary material for: Deep sampling of the Palomero maize transcriptome by a high throughput strategy of pyrosequencing
Source: BMC Genomics. 2009 Jul 6;10:299. doi: 10.1186/1471-2164-10-299 (PMC2714558; doi:10.1186/1471-2164-10-299)
Supplement: Additional file 3 — Primers used in this study. This table contains the sequence of primers used for qRT-PCR. [file 1471-2164-10-299-S3.doc]

**Additional file 3. Primers used in this study.**

| **Primer name** | **Sequence 5’ to 3’** |
| --- | --- |
| **TC327885-for** | **TCGCAGCTGACACAACCCAAGAC** |
| **TC327885-rev** | **CGCACGCCTGAAGACCCTGAT** |
| **TC361477-for** | **AGAGGGGCGCCAAGGTGAAGA** |
| **TC361477-rev** | **CCGGCCGACCCCACTCATT** |
| **TC369342-for** | **GACGGACGCACACTTGCTGACTAC** |
| **TC369342-rev** | **TCCTGGATCTTTGCCTTGACATTG** |
| **TC326717-for** | **TTCTCACGCATCGACCACAAGTTT** |
| **TC326717-rev** | **GTCTCCCTCGTCACCCTCATCATC** |
| **TC342043-for** | **CTTTGCTGCACGGGAGGAATG** |
| **TC342043-rev** | **ATGGACGCACGCTGGCTGACTA** |
| **TC364641-for** | **AACGCCGGACTGGAAATTG** |
| **TC364641-rev** | **TGTTCGGACTCGGAGGTGG** |
| **TC327155-for** | **CGTCTTCGGCGACACTACCA** |
| **TC327155-rev** | **TTACTTCTACCTCTACGTCTTCACAA** |
| **TC327230-for** | **GCGATGATGAGAAAGGGCAAT** |
| **TC327230-rev** | **GAATGGCACACGGAGTAGGC** |
